# Scarlatiniform Rash Caused by Mycoplasma pneumoniae

**DOI:** 10.7759/cureus.8881

**Published:** 2020-06-28

**Authors:** Arooj Mohammed, Sahand Rahnama-Moghadam

**Affiliations:** 1 Miscellaneous, Indiana University School of Medicine, Indianapolis, USA; 2 Dermatology, Indiana University School of Medicine, Indianapolis, USA

**Keywords:** mycoplasma pneumonia, scarlatiniform rash, pastia’s lines, doxycycline, molecular mimicry, infection

## Abstract

Mycoplasma pneumoniae is a significant cause of acute respiratory disease in children and young adults. We describe a case of a 15-year-old boy who presented with an unusual scarlatiniform rash in the setting of a positive Mycoplasma pneumonia IgM but negative anti-streptolysin antibody. While mycoplasma infections with cutaneous manifestations such as scarlatiniform exanthema have been described in textbooks, there are no reports in the primary literature. We wish to highlight the recognition of alternate presentations of mycoplasma, and briefly discuss the role of molecular mimicry in its pathogenesis.

## Introduction

A 15-year-old boy presented to the dermatology clinic during the transition between summer and fall seasons with a red, diffuse papular rash on his chest, face, arms, and legs. He also displayed photophobia, pulsating headaches, nuchal rigidity, malaise, and myalgias in the upper back and shoulders. A fever with a temperature of 104^o^F (40^o^C) prompted a visit to the ED, where the patient was prescribed clindamycin for possible scarlet fever given the appearance of his rash, but his symptoms did not improve. He continued to have malaise, myalgias, nuchal rigidity, and skin eruption. On questioning, a week or two before the onset of rash and urethritis he had a cough, myalgias, and generalized malaise, though he was still able to go to school. He had a dog that would go outside and had been treated for fleas. He would sometimes go camping but had not since the summer.

## Case presentation

**Figure 1:**
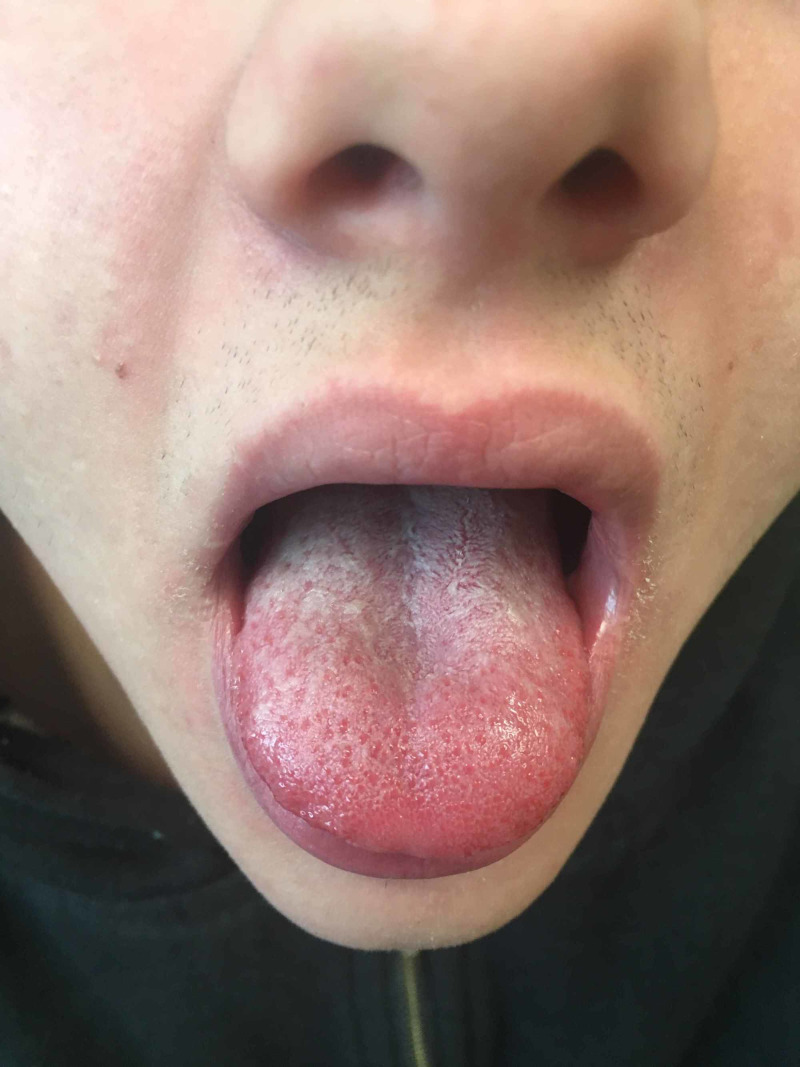
Enlarged fungiform papilla. Enlarged fungiform papilla at an inflamed tongue with white exudate that was able to be scraped off.  Circumoral pallor is present at the upper cutaneous lip.

Inspection and palpation of the skin revealed diffuse plaques of erythema throughout the arms, trunk, and back along with some rough papules. At the tongue, there was white pseudomembranous exudate that was easily wiped away; furthermore there were enlarged fungiform papilla of the tongue and circumoral pallor (Figure 1). 

**Figure 2:**
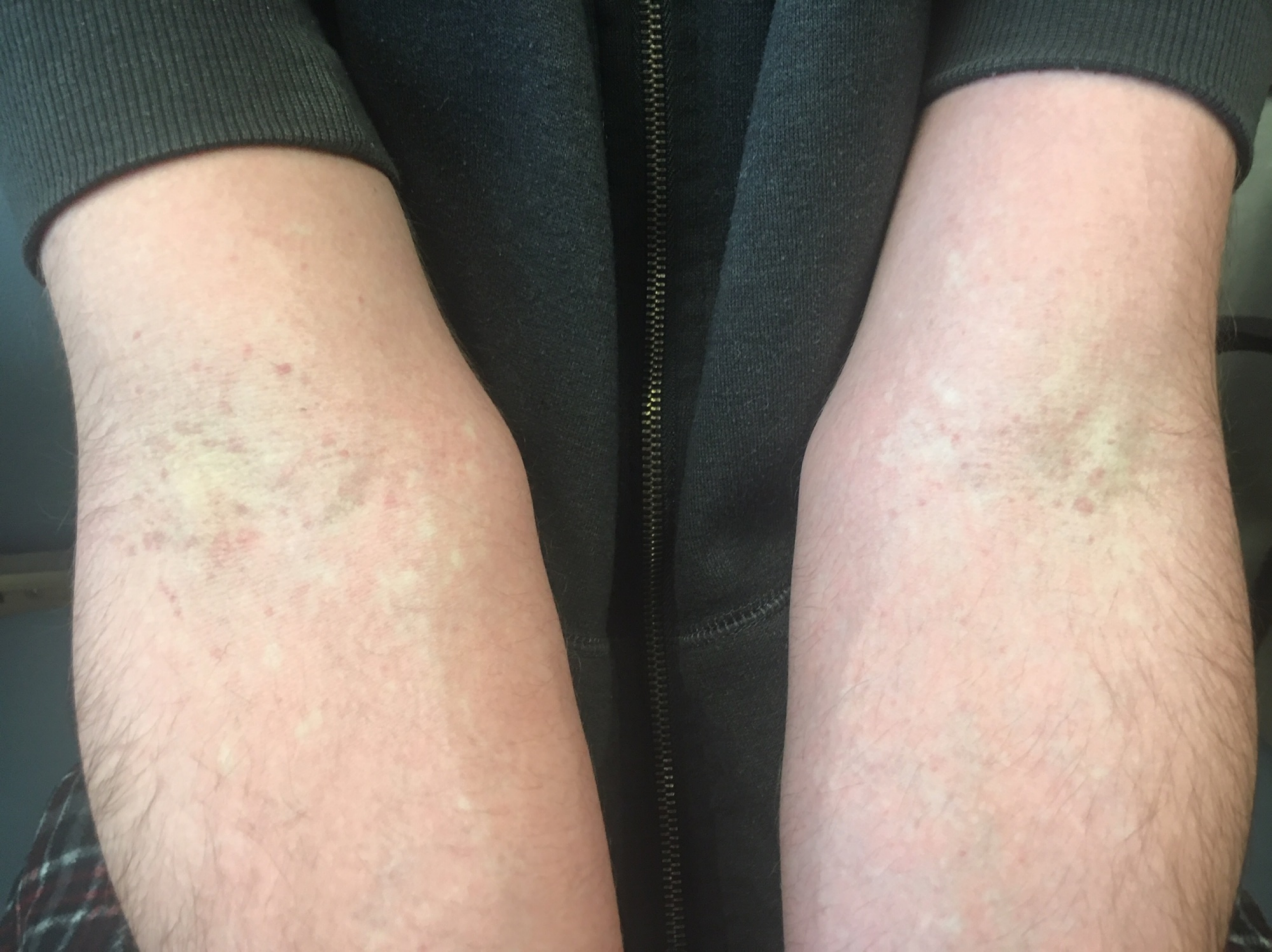
Petechiae. Petechiae at the antecubital fossa—analogous to Pastia’s lines.

At the arms, there was a linear array of petechiae at the antecubital fossa, and scattered petechiae to the lower extremities -- all dull rather than bright red (Figure 2).

Laboratory investigations showed low leukocytes, platelets, sodium, and elevated creatinine and aspartate aminotransferase (AST). Anti-streptolysin antibodies (ASO) were checked to evaluate for scarlet fever and were normal. Blood and throat cultures were also negative. The patient’s constitutional symptoms, myalgias, and headaches that developed acutely during the summer/fall suggested an atypical organism as the cause of infection. Given the patient’s exposure to pets with flea problems, a rickettsial illness such as murine typhus was considered as were atypical pneumonia organisms. However, testing for Q-fever, *Chlamydia pneumonia*, *C. psittaci*, *C. trachomatis*, and rickettsial disease were all negative. Testing for Mycoplasma pneumonia IgM was found positive. Doxycycline 100 mg twice daily was prescribed for a seven-day course. Within 48 hours, the patient’s exanthema, enanthem, and constitutional symptoms had resolved. 

## Discussion

In children and young adults, *Mycoplasma pneumoniae* is a significant cause of acute respiratory disease and may account for an estimated 50% of summer pneumonias [1]. Skin eruptions occur during the course of infection, with the most described being Stevens-Johnson like syndrome (SJS) of Mycoplasma induced rash and mucositis (MIRM). Erythema nodosum and Gianotti-Crosti syndrome have also been reported, as well as isolated mucositis without skin lesions [Fuchs syndrome, or *Mycoplasma pneumoniae*-associated mucositis (MPAM)] [2]. While scarlatiniform mycoplasma infections have been described in textbooks, there are no reports in the primary literature [2]. These varied morphologies may result from the distinct pathophysiology responsible for mucocutaneous diseases associated with Mycoplasma.

Potential mechanisms of Mycoplasma-induced skin disease include direct cytotoxic injury to epithelial cells, immune complex‐mediated vascular injury, or autoimmune attack. Exposure to *M. pneumoniae* is theorized to result in the development of autoantibodies against mycoplasma p1-adhesion molecules, which share extensive sequence homology to mucosal keratinocyte antigens [3-5]. This molecular mimicry is thought to go along with the finding that Mycoplasma has been isolated from the respiratory tract rather than cutaneous lesions in MPAM, supporting an autoimmune response theory over one of direct pathologic effect [6].

Erythromycin, tetracyclines (particularly doxycycline), and fluoroquinolones administered for 7-14 days are equally effective in treating *M. pneumoniae* infections. Tetracyclines are efficacious for most mycoplasmas and chlamydia infections and are the treatment of choice for rickettsial disease [2]. Given the pathophysiology of an autoimmune reaction leading to Mycoplasma associated eruptions, antibiotics may treat an infection but may not alter the course of eruption [6].

## Conclusions

In this case, we aimed to highlight a rare presentation of *M. pneumoniae* as a scarlatiniform rash and discuss the role of molecular mimicry in its pathogenesis. Clinicians should be aware of this unique manifestation and consider this in the differential diagnosis of patients admitted with scarlatiniform rash.
